# Precision implementation of early ambulation in elderly patients undergoing off-pump coronary artery bypass graft surgery: a randomized-controlled clinical trial

**DOI:** 10.1186/s12877-020-01823-1

**Published:** 2020-10-14

**Authors:** Zhaomei Cui, Na Li, Chaonan Gao, Yiou Fan, Xin Zhuang, Jing Liu, Jie Zhang, Qi Tan

**Affiliations:** 1 Intensive Care Unit (ICU), Department of Cardiac Surgery, Shandong Provincial Hospital Affiliated to Shandong First Medical University, Jinan, 250021 Shandong China; 2 Department of Gynecology, Shandong Provincial Hospital Affiliated to Shandong First Medical University, Jinan, 250021 Shandong China; 3 Department of Biostatistics, School of Public Health, Cheeloo College of Medicine, Shandong University, Jinan, 250012 Shandong China; 4 Department of Toxicological and Functional Test, Centers for Disease Control and Prevention of Shandong, Jinan, 250014 China; 5 Intensive Care Unit (ICU), Department of Cardiac Surgery, Shandong Provincial Hospital, Cheeloo College of Medicine, Shandong University, Jinan, 250021 Shandong China

**Keywords:** Early ambulation, Physical rehabilitation, Enhanced recovery after surgery, Cardiac surgery, Elderly patients

## Abstract

**Background:**

Although early ambulation (EA) is associated with improved outcomes in post-operative patients, implementation of EA in elderly patients is still a challenge. In this study, we aimed to design and assess a precision early ambulation program for cardiac rehabilitation.

**Methods:**

We conducted a single-center, randomized and controlled clinical trial in elderly patients aged over 60 years after off-pump coronary artery bypass graft (OPCABG) surgery. Patients were randomly assigned to a precision early ambulation (PEA) group or a routine ambulation (Control) group. Age-predicted maximal heart rate (APMHR) and maximal oxygen uptake (VO_2max)_ were used as a reference to formulate and monitor the PEA regimen. The primary end-point was the postoperative length of stay in hospital (PLOS). The secondary end-points included 90-day mortality, incidence of early discharge, laboratory tests, length of ICU stay, the incidence of multiple organ complications and post-traumatic stress disorder (PTSD). Ambulation outcomes were also recorded.

**Results:**

In total, 178 patients were enrolled (*n* = 89 per group). In the intent-to-treat analysis, PLOS in the PEA group was shorter than that in the Control group (9.04 ± 3.08 versus 10.09 ± 3.32 days, respectively. Mean difference 1.045 days; 95% confidence interval [CI] 0.098–1.992; *P* = 0.031 in the unadjusted model; mean difference 0.957 days; CI 0.007–1.907; *P* = 0.048 in adjusted model). The incidence of early discharge differed significantly between the PEA and control groups (41[46.1%] versus 24[27.0%] patients, respectively. Odds ratio [OR] 0.432; CI 0.231–0.809; *P* = 0.009 in unadjusted model; OR 0.466; CI 0.244–0.889, *P* = 0.02 in adjusted model). The time of first bowel movement, partial pressure O_2_ and post-traumatic stress disorder score in the PEA group were better than those in the Control group. Participants walked much longer distances on day 3 in the PEA group than those in the Control group (76.12 ± 29.02 versus 56.80 ± 24.40 m, respectively, *P* < 0.001).

**Conclusion:**

APMHR and VO_2max_ are valuable for implementation of PEA according to an established security threshold. PEA after OPCAPG surgery is safe and reliable for elderly patients, not only reducing the hospital stay, but also improving their physiological and psychological symptoms.

**Trial registration:**

This study is a component of a protocol retrospectively registered: Application of ERAS in cardiovascular surgery. Trial registration number: ChiCTR1800018167. Date of registration: 3rd September, 2018. URL of trial registry record: http://www.chictr.org.cn/index.aspx

## Background

China faces a tsunami in the aging population, and it is estimated that by the end of 2019, the number of adults aged over 60 years had reached 230 million. By 2050, it is expected that there will be 400 million Chinese citizens aged over 65 years, including 150 million aged over 80 years [1]. The older an individual patient, the higher a surgeon’s threshold is for performing a more extensive or complicated operation [2, 3]. Coronary heart disease is now the leading cause of death, with the mortality in China at 9.2% per year for men and 7.0% for women. Off-pump coronary artery bypass grafting (OPCABG) surgery is regarded to be an ideal approach for elderly patients with coronary heart disease [4, 5]. Despite advances in cardiac surgery resulting in safer procedures, postoperative complications are still frequent, and a determinant of length of hospital stay and functional recovery [6]. It is worth noting that prolonged bed rest is recognized as a well-established contributor to delayed recovery.

Enhanced recovery after surgery (ERAS) is an approach generated from evidence-based medicine that aims to achieve an uneventful recovery after surgery [7, 8]. Early ambulation (EA) following surgery has multiple benefits, enhancing not only recovery of functional exercise capacity and self-perceived functional status, but also muscle force [9]. However, bed rest is prescribed after surgery, with on average, 83% of the patient’s time spent lying in bed, 13% of the time spent seated, and 4% of the time spent walking [10]. Although early mobilization is implemented for patients after cardiac surgery, no consensus exists regarding the optimal intensities, durations and types of EA [11, 12].

Studies of aerobic and resistance exercise have been conducted to maximize physical recovery and minimize the associated side-effects. Hong reported that heart failure patients could not tolerate physical activities at a monitored heart rate of 70% of the age-predicted maximal heart rates (APMHR) [13]. Maximal oxygen uptake (VO_2max_) and its closely related clinical correlate, cardiorespiratory fitness, are key determinants of both elite performance in endurance excise and mortality in the general population, and the physiological mechanisms associated with aging appear to be responsible for a decline in the V̇O_2max_ of older men [14, 15]. However, exercise-based cardiac rehabilitation after surgery has failed to exploit these valuable parameters for postoperative rehabilitation. Therefore, we hypothesized that formulating an individualized security threshold of exercise for elderly patients based on APMHR and VO_2max_ will provide improved ambulation outcomes.

The aim of this study was to design a precision early ambulation (PEA) programs by focusing on the APMHR and VO_2max_ as a security threshold for cardiac rehabilitation and to clarify the specific effects on postoperative length of stay (PLOS) in hospital, postoperative complications, and physiological and psychological functional return.

## Methods

This study was reported in accordance with the CONSORT 2010 Statement using the template for intervention description and replication (TIDieR).

### Trial design and oversight

This single-center, randomized and controlled clinical two-arm trial was conducted at Shandong Provincial Hospital Affiliated to Shandong First Medical University in China. This study was approved by the Ethics Committee of Shandong Provincial Hospital Affiliated to Shandong First Medical University (approval number 2018–239), and as a subset of the protocol registered on the National Clinical Trial Center (registration number ChiCTR1800018167). The overall protocol involved the application of an enhanced recovery after surgery (ERAS) program, which is used to develop perioperative protocols aimed at optimizing patient outcomes and efficient healthcare delivery. ERAS programs are composed of intervention bundles based on the principles of best practice and standardized and consistent healthcare delivery, including early ambulation and adoption of the prone position. Among the interventions we have implemented during routine clinical practice, the precision implementation of early ambulation appeared to be beneficial for patients and the hospital. Therefore, we implemented the precision implementation of early ambulation from our outline registered protocol. All authors affirm that the data and analyses in this trial are accurate and complete, and the trial was conducted in a manner consistent with the study protocol. In this study, quality control and data management were performed by a third party (Shandong Centers for Disease Control and Prevention, China). The statistical analysis was performed by Cheeloo College of Medicine, Shandong University.

### Participants

Consecutive patients were eligible for enrolment if: they were aged at least 60 years, received OPCAPG surgery and cardiac function Grade I–III based on the NYHA classification. Exclusion criteria included acute myocardial infarction 1 week before the operation; comorbidity of severe lung disease, such as chronic obstructive pulmonary disease, and bronchiectasis and received an emergency surgical procedure. Primary withdrawal criteria included repeated occurrence of sudden events. The full list of inclusion and exclusion criteria is shown in Additional file 1. Written informed consent was obtained from each participant or family member before any procedures were conducted.

### Randomization and masking

**Fig. 1:**
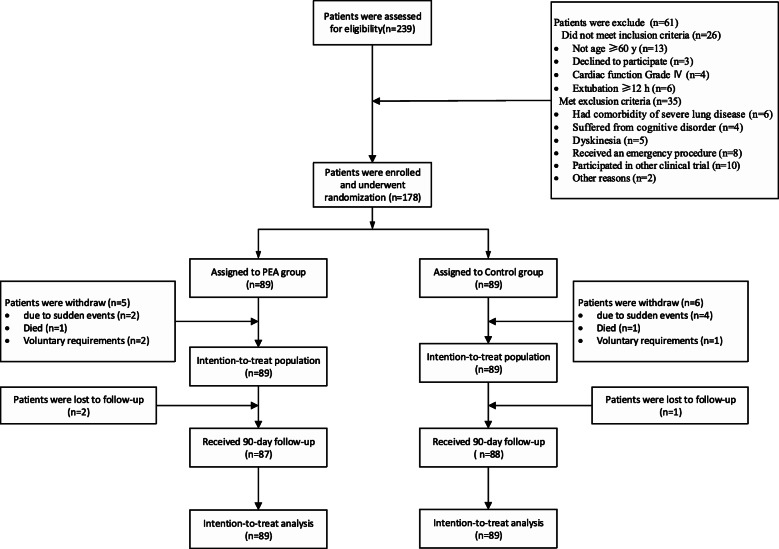
Flowchart showing the patient enrollment procedure including recruitment and random allocation to study group through to follow-up

Patients were assigned to either PEA or routine rehabilitation (Control) group (1:1 ratio) after removal of tracheal intubation, based on the randomization list. Investigators were responsible for confirming eligibility and an independent team was responsible for random group assignment. Group allocation was concealed through a centralized randomization process with a computer-generated randomization list [16]. A centralized randomization process was used, and patients were allocated to groups by an unmasked randomization team that was independent of the study and not involved in recruitment, assessment, or intervention delivery in any way. Due to the nature of the intervention, once the groups of patients were identified, the assignment of treatments was open-label for physicians and patients. However, to mitigate selection bias, data collectors and outcome assessors were kept blinded to study group assignment until study had been completed and the data locked. Details of the protocol for patient randomization are shown in Fig. 1. All patients in the PEA and Control groups performed the same ERAS procedures, with the exception of ambulation (Additional file 1).

### Clinical PEA protocol

The PEA protocol was designed by a panel of experts, including a cardiac surgeon, a rehabilitation therapist, two experienced nurses and two respiratory therapists. The experts formulated a protocol of appropriate exercise intensity and security threshold of heart rate based on the VO_2max_ and APMHR values (Table S1). The PEA protocols were implemented by a group of six rehabilitation therapists with a mean of 11.3 years of experience, with two therapists for every ambulation.

**Fig. 2:**
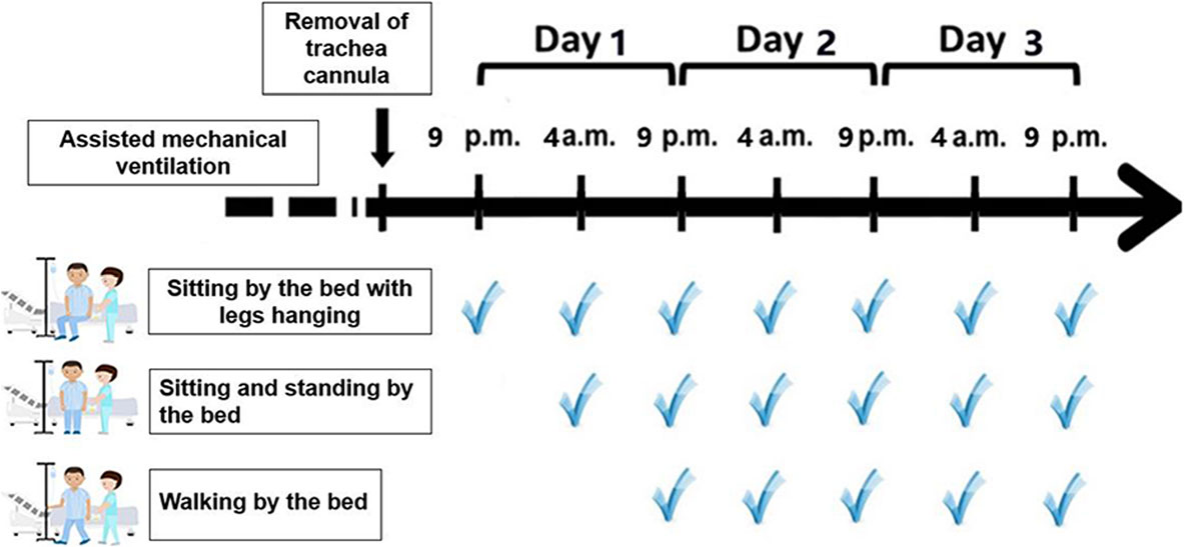
Protocol showing the implementation of precision early ambulation within the first 3 days after surgery

The flowchart of PEA implementation is shown in Fig. 2; the protocol comprised the following steps: 1) On the first day after surgery, patients were assisted to make the transition from sitting up in bed to a seat at the bedside, with their legs hanging down for more than 10 min. If there were not sudden events (Table S2), they were allowed to sit at the bedside or stand for 3–5 min. This process could be repeated less than five times; 2) On the second day after surgery, patients were assisted to sit out of bed. In addition, patients were asked to stand for 3–5 min and for those who were able, to attempt to walk a distance with a minimum target of 20 m with the help of rehabilitation therapists. The PEA protocol was individualized and implemented within the tolerable range. If the maximum HR calculated according to the APMHR and VO_2max_ and other sudden events did not breach the “warning line”(Table S1 and S2), patients were encouraged to engage in more intensive and high-frequency exercise, but no more than five times; 3) On the third day after surgery, patients were assisted to sit out of bed for more than 10 min. In addition, patients were asked to stand for 5 min and attempt to walk a distance with a minimum target of 30 m with the assistance of rehabilitation therapists or family members. Corresponding individualized maximum HR and sudden events were monitored, and patients were encouraged to repeat this process with more intensity and at higher frequency, but no more than five times. 4) On the third day after surgery, patients were encouraged to return to an independent lifestyle and walk greater distances independently as long as sudden events were monitored. If a sudden event occurred during the implementation of any PEA procedure, the rehabilitation therapist had the right to terminate the PEA after the evaluation.

### Routine rehabilitation protocol

The routine rehabilitation protocol was as follows: patients were allowed to engage in ambulation on day 2 or day 3 after surgery. The duration and intensity of ambulation were determined based on the patient’s self-assessment and the experiences of rehabilitation therapists, as long as there were no sudden events.

### Ambulation outcomes and adverse events

The ambulation outcomes of patients in the PEA and Control groups were recorded. The goal of ambulation on day 2 after surgery (Goal-2nd) was to walk approximately 30 ft, while goal on day 3 after surgery (Goal-3rd) was to walk 60 ft. Orthostatic hypotension (OH) and orthostatic intolerance (OI) were monitored closely. OH was characterized by symptoms of dizziness, nausea, weakness, and palpitation, accompanied by a decrease in systolic blood pressure of > 40 mmHg. OI was characterized by symptoms of dizziness, nausea, blurred vision, or syncope due to failed orthostatic cardiovascular regulation, a decrease in arterial pressure, and cerebral hypoperfusion while standing [17].

### End-points of this study

The primary end-point was the PLOS and the secondary end-points were as follows: a) the mortality rate within 90 days after surgery; b) incidence of early discharge defined as PLOS ≤7 days according to Benjamin et al. [18]; c) duration of ICU stay; d) results of laboratory tests including troponin I (TNI) and creatine kinase isoenzyme-MB (CK-MB) (tested at 8:00 pm for the first 3 days) as well as arterial blood gas analysis including partial pressure O_2_ (PO_2_) and partial pressure CO_2_ (PCO_2_) (tested at 8:00 pm for the first 3 days); e) multi-organ function assessment or incidence of postoperative complications, such as time of first bowel, time of urinary retention, time of drainage tube retention, pulmonary atelectasis, pulmonary infection, pleural effusion, OH, OI, acute kidney injury and need for renal-replacement therapy; f) patients’ mental state assessed on the day 5 after surgery using the PTSD Checklist-Civilian (PCL-C) screening scale, as shown in Additional file 1.

### Statistical analysis

The sample-size calculation was based on previous studies on EA in elderly patients undergoing coronary artery bypass grafting (CABG) and scoliosis surgery, which showed that EA reduced the PLOS from 8.11 ± 7.70 days to 5.33 ± 3.02 days, and 10.3 ± 4.6 days to 5.9 ± 1.1 days [19, 20]. We expected a PLOS of 10 days in the Control group and 8 days in the PEA group, with an assumed standard deviation of 3.8. With a two-sided alpha error of 0.05 and an effect size of 0.9, the required sample-size for each group was calculated as 76. With an estimated 15% attrition due to protocol deviation and withdrawal of consent, at least 87 participants per group were recruited.

Data were presented as means and standard deviations (mean ± SD) for normally distributed variables and as medians and interquartile ranges (IQR) for non-normally distributed variables. Categorical data were expressed as absolute number and frequency (n, %). Missing data were uncommon in the data set; therefore, missing measurement data were replaced by the mean value, while missing count data were replaced by the negative results. We assessed the safety and efficacy of two groups in an intention-to-treat population.

Continuous variables were compared using Student’s *t*-test or the Mann–Whitney test. Categorical data were compared using Pearson’s chi-square test (*χ*^2^). Primary and secondary outcomes were analyzed using two models. First, an unadjusted linear regression or logistic regression model was used to explore associations between treatment groups and primary or secondary outcomes for pre-specified analysis. Based on the analysis using an unadjusted model, we created an adjusted linear regression or logistic regression model to test significant associations of treatment groups with outcomes, with baseline values of age, body mass index (BMI) and sex as covariates for post-hoc analysis. The results of laboratory tests after surgery were analyzed by repeated measurement analysis of variance (RM-ANOVA) with time as the within factor (P_w_), PEA or Control group as the grouping factor (P_g_) and interactions identified in the RM-ANOVA (P_i_) with the post-hoc Bonferroni test. Logistic regression modeling was used to explore the risk factors on early discharge for post-hoc analysis. A two-sided *P* < 0.05 was considered to indicate statistical significance. Statistical analyses were performed using SPSS version 25 (SPSS Inc., IBM, Armonk, NY, USA).

## Results

### Enrollment procedure and baseline clinical features

Participants were recruited from September 2018 through to June 2019. A total of consecutive 239 patients who underwent OPCABG were evaluated for their eligibility. Of these, 194 patients underwent randomization; 89 to the PEA group and 89 to the control group (Fig. 1). Patients were excluded from the study if they had comorbidity of severe lung disease (6/35[17%]), suffered from a cognitive disorder (4/35[11.4%]), dyskinesia (5/35[14.3%]), received an emergency surgical procedure (8/35[22.9%]), or participated in another clinical trial (10/35[28.6%]). In addition, five patients in the PEA group and six patients in the Control group withdrew after allocation, and two patients in the PEA group and one patient in the Control group were lost to follow-up. The basic clinical characteristics of the patients in the PEA and Control groups showed no significant differences (Table 1). All surviving patients were followed-up for 90 days.

**Table 1:** Baseline of patients in PEA and Control group

	PEA group (*n* = 89)	Control group (*n* = 89)	*P*-value
Age (years)	65.1 ± 4.6	66.2 ± 4.5	0.118
Female patients (%)	24 (27.0)	27 (30.3)	0.740
BMI (kg/m^2^)	25.8 ± 3.2	25.8 ± 2.6	0.908
Medical history			
Hypertension (%)	19 (21.3)	30 (33.7)	0.185
Diabetes (%)	13 (14.6)	21 (23.6)	0.181
Renal insufficiency (%)	2 (2.2)	5 (5.6)	0.444
Cerebral infarction (%)	10 (11.2)	6 (6.7)	0.443
Smoking (%)	29 (32.6)	27 (30.3)	0.875
Preoperative ejection fraction (%)	59.2 ± 3.3	58.8 ± 3.7	0.536
Euro Score	7.61 ± 2.14	7.79 ± 2.34	0.453
Number of heart bypasses	4.10 ± 0.45	4.11 ± 0.44	0.465

Note: Data represent the mean ± standard deviation (SD) for continuous variables, and number of patients (n) and percentage (%) for categorical variables. *BMI* body mass index

### Ambulation outcomes and adverse events

As shown in Table 2, a total of 75 (84.3%) patients in the PEA group and 62 (69.7%) in the Control group achieved Goal-2nd. In addition, 74 (83.1%) patients in the PEA group and 49 (55.1%) in the Control group completed Goal-3rd. Within the first 3 days post-surgery, eight (9.0%) patients in the PEA group and 15 (16.9%) in Control group had OH. Furthermore, two (2.2%) patients in Control group had OI, but none in the PEA group. The distance of ambulation on day 3 after surgery was significantly greater in the PEA group than that in the Control group (75.67 ± 29.73 versus 56.17 ± 25.0 m, *P* < 0.001).

**Table 2:** Ambulation outcomes in patients in the PEA and Control groups

	PEA group (*n* = 89)	Control group (*n* = 89)	*P-*value
Goal-2nd	75 (84.3%)	62 (69.7%)	0.021
Goal-3rd	74 (83.1%)	49 (55.1%)	<0.001
The distance of ambulation on day 3 post-surgery	75.67 ± 29.73	56.17 ± 25.0	<0.001
Orthostatic hypotension	8 (9.0%)	15 (16.9%)	0.179
Orthostatic intolerance	0 (0%)	2 (2.2%)	0.497

Note: Data represent the mean ± standard deviation (SD) for continuous variables, and number of patients (n) and percentage (%) for categorical variables

### Primary and secondary end-points of this study

As primary end-point of this study, PLOS was significantly shorter in the PEA group than that in the Control group (9.04 ± 3.08 versus 10.09 ± 3.32 days. Estimate 1.045; 95% confidence interval [CI] 0.098–1.992; *P* = 0.031 in unadjusted model; Estimate 0.957; CI 0.007–1.907, *P* = 0.048 in adjusted model) (Table 3 and Table S3). With respect to secondary outcomes, the time of first bowel movement in the PEA group was significantly earlier than that in the Control group (3.18 ± 1.23 versus 3.97 ± 1.26 days. Estimate 0.787; CI 0.419–1.154; *P* < 0.001 in unadjusted model; Estimate 0.795; CI 0.422–1.169; *P* < 0.001 in adjusted model). Remarkably, the incidence of early discharge was 46.1% (41/89) in the PEA group and 27.0% (24/89) in the Control group (odds ratio [OR] 0.432; CI 0.231–0.809; *P* = 0.009 in unadjusted model; OR 0.466; CI 0.244–0.889; *P* = 0.02 in adjusted model) (Table S4). However, there were no significant differences between the PEA and Control groups in terms of ICU stay, time of drainage tube retention and time of urinary catheter retention (2.98 ± 1.40 versus 3.10 ± 1.50 days, 3.82 ± 0.92 versus 3.89 ± 0.82 days, and 3.00 ± 1.02 versus 3.22 ± 0.88 days, respectively, all *P* > 0.05). There were also no significant differences in the incidence of atelectasis (12.4% versus 19.1%, *P* > 0.05) and pulmonary infection (12.4% versus 14.6%, *P* > 0.05) between the two groups. In terms of the results of laboratory tests, the PO_2_ values at different time-points in the PEA group were better than those in the Control group (*P*_*g*_ = 0.001), while there were no significant differences in TNI and CK-MB (*P*_*g*_ *=* 0.599*, P*_*g*_ *=* 0.415, respectively) between the two groups (Table S5). As shown in Table S6 and Table 4**,** univariate and multivariate analyses confirmed an association between early discharge and PEA (*P* = 0.024), age (*P* = 0.009), Euro score (*P* = 0.008) and cerebral infarction (*P* = 0.049). Furthermore, the PTSD score was much lower in the PEA group than that in the Control group (27.72 ± 9.34 versus 40.44 ± 12.55, respectively, *P* < 0.001) (Table S7).

**Table 3:** Comparative analysis of the primary and secondary study end-points in patients of the PEA and Control groups ^a,b^

	PEA group (*n* = 89)	Control group (*n* = 89)	*p-*value
**Primary end-point**			
PLOS (days)	9.04 ± 3.08	10.09 ± 3.32	0.031
**Secondary end-point**			
Mortality within 90 days (%)	1 (1.1)	1 (1.1)	1.000
Incidence of early discharge	41 (41.6)	24 (27)	0.009
Duration of ICU stay (days)	2.98 ± 1.40	3.10 ± 1.50	0.570
Time of drainage tube retention (days)	3.82 ± 0.92	3.89 ± 0.82	0.648
Time of first bowel movement (days)	3.18 ± 1.23	3.97 ± 1.26	<0.001
Time of urinary catheter retention (days)	3.00 ± 1.02	3.22 ± 0.88	0.129
Incidence of acute kidney injury (%)	6 (6.7)	6 (6.7)	1.000
Number of patients with renal-replacement therapy	0	1 (1.1)	0.323
Incidence of pulmonary atelectasis (%)	11 (12.4)	17 (19.1)	0.174
Incidence of pulmonary infection (%)	11 (12.4)	13 (14.6)	0.613

Note: Data represent the mean ± standard deviation (SD) for continuous variables, and number of patients (n) and percentage (%) for categorical variables

*PLOS* postoperative length of stay in hospital;

^a^: simple linear regression (unadjusted)

^b^: Missing data: on pulmonary atelectasis for two patients in the PEA group and two patients in the Control group

**Table 4:** Baseline factors predictive of early discharge based on a multivariate logistic regression analysis

Variable	OR	95%CI	*P-*value
Randomization to PEA	0.461	0.235–0.904	0.024
Age	0.901	0.833–0.974	0.009
Euro score	0.753	0.61–0.929	0.008
Cerebral infarction	0.201	0.041–0.092	0.049
Sex	0.901	0.376–1.784	0.616
BMI	0.938	0.835–1.054	0.279

Note: *BMI* body mass index; *PEA* precision early ambulation; *OR* odds ratio; *CI* confidence interval

## Discussion

To the best of our knowledge, this is the first trial of the formulation of early ambulation programs with precision intensity, duration and individualized security thresholds based on APMHR and VO_2max_ in elderly patients after cardiac surgery. By individualized implementation of this protocol, significant and favorable associations were found to exist between PEA and clinical outcomes, such as PLOS. Multivariate logistic regression analyses confirmed the effect of PEA on early discharge.

The characteristics of the participants in this study were consistent with the predominance of coronary heart disease in elderly patients. It was commonly noted that aged patients undergoing CABG were at a higher risk of perioperative complications and death, and that EA was difficult to be implement in this population. Our data showed that advanced age was an independent risk factor for early discharge. Frustratingly, there is no real consensus among clinicians regarding the definition and implementation of “early” in elderly patients. Few studies provided a clear definition of EA after cardiac surgery. David reported that the gradual increase in activity should be started on the first postoperative day until independent ambulation on the fifth postoperative day [21]. However, Jans et al. reported that EA might lead to a high incidence of postoperative OH and OI, especially in the elderly population [22]. We recommend that a progressive program, starting with standing by the bed within 24 h and walking after 24 h was safe and helpful for function recovery. Our findings appear to support the importance of avoiding bed rest for the safety of elderly patients after cardiac surgery.

EA should constitute a continuum of care and multiple therapy techniques. The Heart Failure Quality Program define EA as the patient’s ambulation without assistance [23]. In some superior ICUs, patients engage in novel mobilization techniques, such as cycle ergometer, upper body exercises, Kinarm robotic exoskeleton and supported treadmill training [24, 25]. Andrew et al. reported that walking provided a well-tolerated and clinically effective alternative to stationary cycling in the early postoperative period after CABG [26]. However, there is no evidence that one technique is superior to another. Another important factor regarding early mobilization is the intensity of EA. Moradian et al. showed that patients who received low-frequency monitored exercise programs had higher discharge rates and shorter ICU stay [27]. The expected goal in another study was 100 m of walking with assistance on day 2 after CABG, and a 10-min physiotherapy-supervised walking exercise session on day 3 [26]. Our data showed that 83% patients completed a low-intensity exercise implemented in a step-by-step process on day 3 after surgery with a low incidence of OH and OI, which was much better than that observed in the Control group. According to our clinical experience, excessive exercise is more of a hindrance than a help. Therefore, exercise physiology values such as APMHR and VO_2max_ are implicated as novel and efficient “warning lines” for physiological responses [13, 28]. We believe low-frequency exercises based on VO_2max_ and APMHR monitoring could be beneficial and accurately reflect an elderly patient’s tolerance in the early postoperative period while guaranteeing their safety.

Future efforts directed at evaluating perioperative care for elderly patients will need to be comprehensive and include traditional outcome measures, such as mortality and complications, as well as focusing more attention on quality measures, such as PLOS and readmission. Shorter hospital stays lead to benefits for patients, their families, and the hospital, contributing to net healthcare costs savings. However, prolonged hospital stay after surgery predisposes patients to clinical complications and increases medical costs. There is growing evidence that early mobilization of patients in the ICU can reduce the length of hospital and ICU stays [29]. Cacau et al. reported that an early mobility protocol decreased the length of stay from 12.2 days to 9.4 days [30]. Schaller et al. reported that the length of ICU/hospital stay was 7/15 days in the early intervention group and 10/21.5 days in the control group, respectively [31]. Therefore, it is understandable that the length of hospital stay is the outcome most frequently used to assess the effectiveness of early mobilization in elderly patients. However, in contrast with these results, some other trials did not identify this effect of early mobilization. Klein noted that there was insufficient evidence on the effect of early mobilization of critically ill people in the ICU on physical function or performance, adverse events, muscle strength and health-related quality of life at this time [32]. Although our results are consistent with the previous findings that PEA shortens the length of PLOS stay, we showed conflicting results in that the length of ICU stay was reduced by EA. We speculated that the mainly elderly population of patients enrolled in our study faced a slower early recovery as a result of organic aging during the period in the ICU. Factors affecting early discharge destination in elderly patients is potentially confounded by multiple factors. Some studies showed that a higher level of independence in activities of daily living or less degree of frailty was significantly related to early discharge among younger patients, better functional and cognitive status [33, 34]. In our study, we found that EA was an independent factor that was as important as age and Euro score in achieving an early discharge.

EA is also beneficial in terms of functional capacity and prevention of postoperative complications. Li et al. reported that ERAS effectively brought forward the time of first bowel movement by approximately 1.0 day [11]. Our data also confirmed that PEA promoted gastrointestinal function recovery, with an average reduction in the time of first bowel movement to 0.7 day. In our study, the incidence of pulmonary atelectasis and pulmonary infection in the PEA group was reduced by 7 and 2%, respectively. In contrast, Moradian et al. showed that EA reduced the incidence of pleural effusion and atelectasis by approximately 34 and 29%, respectively [27]. However, arterial oxygen level was significantly improved in the PEA group, suggesting that early ambulation could contribute to lung recovery, and improvement of circulatory function.

Growing evidence highlights that positive affectivity is associated with better cardiac rehabilitation adherence and plays a role as an independent factor influencing cardiac outcomes. Approximately 20–51% of patients with coronary heart disease are affected by clinical psychological symptoms [35]. PTSD is an abnormal psychological reaction characterized by a series of anxious manifestations such as avoidance, and a state of high alert. Gao et al. have proposed that 25.8% of patients with myocardial infarction had PTSD [36]. Deng et al. reported that the incidence of post-operative PTSD in 134 adults with congenital heart disease was 21% [37]. Our study confirmed a strong negative association of PTSD with EA, which indicated that positive psychological functioning, such as positive attitude and optimism, stimulates goal-striving activities that encourage protective health behaviors and to adherence to therapies.

Our study has some limitations. First, there was a low power of the study for the secondary endpoint due to the small sample size and clinical limitation of early ambulation. Therefore, a larger sample size should help to increase the power. Second, the narrow inclusion criteria of age and type of surgery applied in this study may increase the risk of bias due to selective reporting. Broader inclusion criteria will help to increase the generalizability of our results. However, clinical experience shows that ERAS may be more beneficial for aged patients, and cardiopulmonary bypass in cardiac surgery may have a potential negative impact on the recovery of patients. Therefore, we enrolled elderly patients undergoing OPCABG to identify significant clinical differences. Third, a blinded randomized controlled trial would provide more robust evidence; however, it was impossible to blind participants and therapists from the intervention in our study and we could not avoid the subjective implementation of the EA protocols by rehabilitation therapists and nurses in this study. Fourth, although early ambulation is an important part of ERAS, the clinical effect of early ambulation is not only determined by the effectiveness of the intervention, but also by the quality of the usual EA procedure and the clinical skill of the rehabilitation therapists as well as patient compliance in different hospitals. This study was a component of a protocol registered for ERAS that included several interventions such as respiratory exercise, acupuncture, and early ambulation. The interaction of multiple interventions has produced variable phenotypes of recovery of patients. This fact also limits the generalizability of the results to other hospitals with different standards of routine care. Fifth, although missing data were uncommon in this study, the chosen strategy of replacement of missing values with the mean value and negative results also may decrease the variability and act as a bias that decreases differences.

## Conclusion

In summary, our data confirmed that PEA can shorten the PLOS, reduce postoperative complications, and accelerate physiological and psychological rehabilitation of elderly patients after OPCABG surgery. Our study also revealed that APMHR and VO_2max_ are valuable for implementation of PEA according to an established security threshold. Further investigations are required to improve the formulation and implementation of EA by focusing on factors such as cross-disciplinary integration, systematic training and individualized treatment.

## Supplementary information

**Additional file 1.** Supplemental description of methods and results, including study eligibility criteria, rehabilitation measure, PTSD Checklist-Civilian (PCL-C) screening scale, and Tables S1–S7.

## Data Availability

The datasets used and/or analyzed during the current study are available from the corresponding author on reasonable request.

## Abbreviations

EA: Early ambulation
OPCABG: Off-pump coronary artery bypass graft
PEA: Precision early ambulation
APMHR: Age-predicted maximal heart rate
PLOS: Postoperative length of stay
PTSD: Post-traumatic stress disorder
PO_2_: Pressure O_2_
PCO_2_: Partial pressure CO_2_
OH: Orthostatic hypotension
OI: Orthostatic intolerance
CABG: Coronary artery bypass grafting
ICU: Intensive care unit
ERAS: Enhanced recovery after surgery
TNI: Troponin I
CK-MB: Creatine Kinase isoenzyme-MB
