# Supplementary material for: Precision implementation of early ambulation in elderly patients undergoing off-pump coronary artery bypass graft surgery: a randomized-controlled clinical trial
Source: BMC Geriatr. 2020 Oct 14;20:404. doi: 10.1186/s12877-020-01823-1 (PMC7560239; doi:10.1186/s12877-020-01823-1)
Supplement: Supplementary file 1 — Additional file 1. Supplemental description of methods and results, including study eligibility criteria, rehabilitation measure, PTSD Checklist-Civilian (PCL-C) screening scale, and Tables S1–S7. [file 12877_2020_1823_MOESM1_ESM.docx]

**Supplementary Appendix**

This appendix has been provided by the authors to provide readers with additional information about this study.

**Precision implementation of** **early ambulation in elderly patients** **undergoing off-pump coronary artery bypass graft surgery: a randomized controlled clinical trial**

Zhaomei Cui ^1#^, Na Li ^3#^, Chaonan Gao^4^, Yiou Fan^5^, Xin Zhuang^1^, Jing Liu^1^, Jie Zhang^1^, Qi Tan^1,2^**^*^**

**TABLE OF CONTENTS**

Study eligibility criteria page 2

Rehabilitation measure page 3

PTSD Checklist-Civilian (PCL-C) screening scale page 3

**Table S1.** The formula for calculation of APMHR and VO_2_max of patients in the PEA group.

page 5

**Table S2.** Withdrawal criteria for adverse events. page 4

**Table S3.** Linear regression of primary and some secondary outcomes as dependent variables

page 6

**Table S4.** Logistic regression of some secondary outcomes as dependent variables page 7

**Table S5.** Laboratory tests of patients in the PEA and Control groups.

page 8

**Table S6.** Univariate analysis of associations between baseline variables and early discharge.

page 9

**Table S7.** Comparison of PTSD score in patients in the PEA and Control groups. page 10

***Study eligibility criteria***

Diagnosis and main criteria for inclusion, exclusion and withdrawal:

Primary inclusion criteria:

(I) age ≥60 years; (II) received OPCAPG surgery; (III) had cardiac function Grade I–III based on the NYHA classification; (IV) tracheal intubation removed within 12 h after OPCABG surgery; (V) provided informed consent.

Primary exclusion criteria:

(I) had acute myocardial infarction one week before the operation; (II) with comorbidity of severe lung disease, such as chronic obstructive pulmonary disease, and bronchiectasis; (III) received an emergency surgical procedure; (IV) suffered from cognitive disorder or refused to cooperate; (V) dyskinesia due to nervous system disorder or trauma; (VI) participated in other clinical trials.

Primary withdrawal criteria:

(I) repeated occurrence of sudden events (**Table S2),** such as unpredictable emergencies more than four times cumulatively or more than twice during a single ambulation; (II) hemoglobin (Hb) <70 g/L or drainage volume >1,000 ml within 12 h after surgery; (III) voluntary withdrawal; (IV) could not complete the study due to serious organ dysfunction, such as cerebral infarction, cerebral hemorrhage, renal failure and death.

***Rehabilitation measure***

In addition to different ambulation protocols, similar Enhanced recovery after surgery (ERAS) procedures were implemented for patients in the PEA and Control groups. Preoperative measurements included preoperative assessment, education and psychological counselling, respiratory exercise, and antibiotic prophylaxis within 30 min of the start of anesthesia. Intraoperative measurements included lung protection strategies, blood conservation measurements, cerebral oxygen saturation monitoring, Swan–Ganz monitoring and insulation intervention. Postoperative measurements included analgesia, nausea and vomiting prevention, inspiratory muscle training, early food intake and early removal of drainage tube. The precondition for ambulation in the two groups were as follows: 1) patients with clear consciousness, emotional stability, and could accurately answer open questions; 2) electrocardiograph monitoring: blood pressure 90–160/60–100 mmHg, heart rate ≥50 beats/min, and pulse oxygen saturation (SpO_2_) >92%, partial pressure O_2_ (PaO_2_) >50 mmHg; 3) drainage volume <200 ml within 4 h before getting out of bed; 4) muscle strength assessment ≥3 ^[^[^17^](#_ENREF_17)^]^; 5) administration of dopamine ≤2 μg/kg·min.

***PTSD Checklist-Civilian (PCL-C) screening scale***

The PCL-C included 17 items, each of which had five levels, with a maximum total score of 85. The PCL-C scores were assigned according to the following criteria: 17–37 points: normal, no obvious symptoms of PTSD; 38–49 points: mild, a certain degree of PTSD symptoms; 50–85 points: moderate/severe, obvious symptoms of PTSD.

**Table S1.** The formula for calculation referring APMHR and VO_2max_ values for patients in the PEA group.

| Items | ^a^ Calculation |
| --- | --- |
| The maximal exercise HR on day 1 (% MHR) | 0.64×10+37–43% |
| The maximal exercise HR on day 2 (% MHR) | 0.64×20+37–50% |
| The maximal exercise HR on day 3 (% MHR) | 0.64×30+37–56% |
| HRR (bpm) | 205.8-0.685×60=165 |
| 47% of APMHR ^a^ (bpm) | 165×43%+50=121 |
| 50% of APMHR (bpm) | 165×50%+50=132 |
| 56% of APMHR (bpm) | 165×56%+50=142 |

Note: VO_2max_ was set as 10% on day 1, 20% on day 2 and 30% on day 3 after surgery.

^a^ Formula[6]:

%MHR = 0.64 × %VO_2max_+ 37; HR_max_=205.8-0.685×age;

HRR=HR_max_-RHR; X% APMHR=HRR×X%+RHR

*Abbreviations in the above formula:*

APMHR: Age predictive maximum heart rate; SpO_2_: Surplus pulse O_2_;

%MHR: Maximum exercise heart rate; %VO_2max_: Maximum oxygen uptake;

HR_max_: Maximum heart rate; HRR: Heart rate reserved;

RHR: Resting heart rate.

**Table S2.** Withdrawal criteria for sudden events.

| **HR** | **SpO_2_ and RR** |
| --- | --- |
| Ventricular arrhythmia or conduction block >Grade II  Decreased by >20% compared to resting state  <40 beats per min (bpm) or >130 bpm  >Max HR calculated by APMHR and VO_2max_ ^a^ | Decreased SpO_2_ by >6% compared to resting state  SpO_2_ <88%  RR >40 times/min |
| **BP** | **Mental state** |
| Systolic pressure (SP) >180 mmHg | RASS score <-3 or >2 |
| Reduction in systolic/diastolic pressure (SP/DBP) >20% | Exertional dyspnea  Unable to tolerate activity |
| Orthostatic hypotension or orthostatic intolerance |  |
| Dopamine dosage >5 µg/kg·min |  |

Note: HR: Heart Rate; SpO2: pulse oxygen saturation; RR: respiratory rate; BP: blood pressure; RASS: [Richmond Agitation–Sedation Scale](http://www.baidu.com/link?url=CbmRzMktzRdUK-n8ARCcE3RetBq-GHIri7n_3seN3VH3ao3H3IJj5u6kB_mW7_aHET3iXAbMEqRvo-w1qLLzihKt4K7A1hmMjfxJVq7iM0G).

^a^ Formula for max. heart rate in early ambulation calculated by APMHR and VO_2max_ refers to Table S1

**Table S3.** Linear regression using upright position as the dependent variable. Regression coefficients β, 95% confidence interval (CI) for regression coefficients and *P*-value in the unadjusted and adjusted regression models

| Independent variable | Unadjusted ^a^ | | | Adjusted ^b^ | | |
| --- | --- | --- | --- | --- | --- | --- |
|  | Regression coefficient β | | | Regression coefficient β | | |
|  | Estimate | 95%CI | *P*-value | Estimate | 95%CI | *P*-value |
| Primary end-point |  |  |  |  |  |  |
| PLOS | 1.045 | 0.098, 1.992 | 0.031 | 0.957 | 0.007, 1.907 | 0.048 |
| Secondary end-point |  |  |  |  |  |  |
| Duration of ICU stay | 0.124 | -0.305, 0.553 | 0.57 | 0.079 | -0.349, 0.506 | 0.717 |
| Time of drainage tube retention | 0.067 | -0.191, 0.326 | 0.607 | 0.06 | -0.2, 0.321 | 0.648 |
| Time of first bowel movement | 0.787 | 0.419, 1.154 | ˂0.001 | 0.795 | 0.422, 1.169 | ˂0.001 |
| Time of urinary catheter retention | 0.117 | -0.057, 0.506 | 0.117 | 0.219 | -0.065, 0.503 | 0.129 |

Note: PLOS: postoperative length of stay in hospital;

^a^ Unadjusted model of linear regression; ^b^ Adjusted model of linear regression by age, BMI and sex included as covariates.

**Table S4.** Logistic regression in upright position as dependent variable. Regression coefficients OR, 95% confidence interval (CI) for regression coefficients and p-value in the unadjusted and adjusted regression model

| Independent variable | Unadjusted ^a^ | | | | Adjusted ^b^ | | |
| --- | --- | --- | --- | --- | --- | --- | --- |
|  | Regression coefficient OR | | | | Regression coefficient OR | | |
|  | Estimate | 95%CI | | *P*-value | Estimate | 95%CI | *P*-value |
| Mortality within 90 days (%) | 1 | 0.062, 16.241 | 1 | | 1.475 | 0.062, 34.900 | 0.810 |
| Incidence of early discharge | 0.432 | 0.231, 0.809 | 0.009 | | 0.466 | 0.244, 0.889 | 0.02 |
| Incidence of acute kidney injury (%) | 1 | 0.310, 3.228 | 1 | | 1.044 | 0.319, 3.420 | 0.943 |
| Incidence of pulmonary atelectasis (%) | 1.674 | 0.735, 3.814 | 0.22 | | 1.787 | 0.774, 4.126 | 0.174 |
| Incidence of pulmonary infection (%) | 1.213 | 0.512, 2.875 | 0.661 | | 1.225 | 0.520, 3.025 | 0.613 |

Note: OR: odds ratio;

^a^ Unadjusted model of logistic regression; ^b^ Adjusted model of logistic regression by age, BMI and sex included as covariates.

**Table S5**. Laboratory tests of patients in the PEA and Control groups ^a^

|  | PEA group  (n = 89) | Control group  (n = 89) | *P*-value | *P_W_* | *P_g_* | *P_i_* |
| --- | --- | --- | --- | --- | --- | --- |
| **TNI (ng/ml)** | | | |  |  |  |
| Before surgery | 0.04(0.02, 0.06) | 0.04(0.03, 0.06) | 0.634 |  |  |  |
|  |  |  |  | 0.002 | 0.599 | 0.601 |
| Day 1 after surgery | 0.38(0.25, 0.88) | 0.48 (0.31, 0.86) | 0.207 |  |  |  |
| Day 2 after surgery | 0.30(0.16, 0.51) | 0.31 (0.18, 0.60) | 0.515 |  |  |  |
| Day 3 after surgery | 0.12(0.07, 0.33)^*^ | 0.16 (0.09, 0.39)^*^ | 0.295 |  |  |  |
| **CK-MB (ng/ml)** | | | |  |  |  |
| Before surgery | 1.10(0.50, 1.50) | 1.0(0.50, 1.30) | 0.215 |  |  |  |
|  |  |  |  | ˂0.001 | 0.415 | 0.608 |
| Day 1 after surgery | 4.07(3.00, 6.35) | 4.61 (3.21, 6.77) | 0.348 |  |  |  |
| Day 2 after surgery | 2.55(1.74, 4.98) | 3.15 (1.78, 5.06) | 0.482 |  |  |  |
| Day 3 after surgery | 1.93(1.15, 3.09)^*^ | 1.81 (1.25, 3.29)^*^ | 0.903 |  |  |  |
| **PO_2_ (mmHg)** | | | |  |  |  |
| Before surgery | 76.5±10.0 | 75.9±7.5 | 0.559 |  |  |  |
|  |  |  |  | ˂0.001 | 0.001 | 0.063 |
| Day 1 after surgery | 90.5±20.1 | 84.4±21.7 | 0.055 |  |  |  |
| Day 2 after surgery | 79.6±19.5 | 75.3±18.2 | 0.048 |  |  |  |
| Day 3 after surgery | 84.8±20.2 | 78.2±14.9 | 0.010 |  |  |  |
| **PCO_2_ (mmHg)** | | | |  |  |  |
| Before surgery | 38.4±4.0 | 37.7±3.4 | 0.183 |  |  |  |
|  |  |  |  | ˂0.001 | 0.102 | 0.785 |
| Day 1 after surgery | 38.7±4.1 | 38.0±3.4 | 0.121 |  |  |  |
| Day 2 after surgery | 39.2±4.0 | 38.4±4.2 | 0.114 |  |  |  |
| Day 3 after surgery | 37.3±4.9 | 36.8±4.2 | 0.475 |  |  |  |

Note: Data represent the mean ± SD and median (range) or parameter counts (n). Mann–Whitney U-tests were used to compare TNI and CK-MB, while Student’s *t*-test was used to compare PO_2_ and PCO_2_ at same time-point. TNI, CK-MB, PO_2_ and PCO_2_ at 3 different time-points (day 1 after surgery, day 2 after surgery and day 3 after surgery) were analyzed by repeated-measures analysis of variance (RM-ANOVA). *P*_w_, *P*_g_, *P*_i_ values for within subjects, between groups and for interaction from RM-ANOVA, respectively. ^*^ day 1 vs. day 3 *P*˂0.001.PEA: precision early ambulation

^a^ Missing data: TNI and CK-MB at day 2 for one patient in the control group; TNI and CK-MB at day 3 for two patients in the PEA group and one patient in the control group; pO_2_ and pCO_2_ at day 3 for one patient in the PEA group and three patients in the control group.

**Table S6.** Univariate analysis of association between baseline variables and early discharge.

Any value with *P* < 0.1 was incorporated into a multivariate logistic regression model.

| Variable | Early discharge: YES (n = 65) | Early discharge: NO (n = 113) | *P-*value |
| --- | --- | --- | --- |
| Age (months) | 64.31±4.31 | 66.44±4.60 | 0.003 |
| Female sex n(%) | 15(22.7%) | 36(32.1%) | 0.180 |
| BMI, kg/m^2^ | 25.59±2.76 | 25.94±2.98 | 0.442 |
| Randomization to PEA | 41(62.1) | 48(42.9) | 0.013 |
| Hypertension (%) | 19(28.8) | 30(26.8) | 0.773 |
| Diabetes (%) | 14(21.2) | 20(17.9) | 0.582 |
| Renal insufficiency (%) | 1(1.5) | 6(5.4) | 0.203 |
| Cerebral infarction (%) | 2(3.0) | 14(12.5) | 0.033 |
| Smoking (%) | 17(25.8) | 39(34.8) | 0.208 |
| Preoperative ejection fraction (%) | 59.43±2.89 | 58.76±3.80 | 0.187 |
| Number of heart bypasses | 4.11±0.44 | 4.10±0.45 | 0.983 |
| Euro score | 4.02±1.62 | 4.87±1.77 | 0.002 |

Note: Data represent the mean ± standard deviation (SD), and number of subjects (n) and percentage (%) for categorical variables. BMI: body mass index; PEA: precision early ambulation

**Table S7.** Comparison of PTSD scores of patients in the PEA and Control groups.

| Number of patients (%) | PEA group  (n = 89) | Control group  (n = 89) | *P-*value |
| --- | --- | --- | --- |
| Total score | 27.72±9.34 | 40.44±12.55 | ˂0.001 |
| Normal | 70 (78.7%) | 56 (62.9%) |  |
| Mild | 16 (20.0%) | 26 (29.2%) |  |
| Moderate/Severe | 3 (3.4%) | 7 (7.9%) |  |

Note: Data represent the mean ± SD or n (%). PEA: precision early ambulation
